# Engineered Bacteriorhodopsin Film with Oriented Patterns for the Improvement of the Photoelectric Response

**DOI:** 10.3390/ijms232416079

**Published:** 2022-12-16

**Authors:** Mian Wu, Feng Lin, Yu Song

**Affiliations:** 1 Department of Mechanical Engineering, Tsinghua University, Beijing 100084, China; 2 Biomanufacturing and Rapid Forming Technology Key Laboratory of Beijing, Beijing 100084, China; 3 Key Laboratory of Advanced Materials Processing Technology of Ministry of Education, Beijing 100084, China

**Keywords:** bacteriorhodopsin, efficiency factor, energy conversion, immobilization technique, photoelectric response

## Abstract

The use of photosensitive proteins has become a competitive solar energy solution, owing to its pollution-free nature, high conversion efficiency, and good biocompatibility. Bacteriorhodopsin (bR) is an important light-sensitive protein that is widely used in the fabrication of photoelectronic devices. However, research on the optimization and comparison of the immobilization techniques is lacking. In this study, in order to obtain bR films with a high energy conversion efficiency, three immobilization techniques, namely dropcasting, electrophoretic sedimentation, and Langmuir–Blodgett deposition, were used to fabricate films, and their topographical and photoelectrical characteristics were compared. All three immobilization techniques can transfer bR molecules to substrates, forming functional photosensitive bR films. The absorption of the bR films at 568 nm reached the highest value of 0.3 under the EPS technique. The peak photocurrent for the EPS technique reached 5.03 nA. In addition, the EPS technique has the highest efficiency factor of 13.46, indicating that it can generate the highest value of photocurrent under the same light conditions, owing to the improved orientation, and no significant decrease in the peak photocurrent was observed after three weeks, which indicates the stability of the photoelectric response. These results indicate that the EPS technique has a great potential for the photoelectrical device fabrication and solar-energy conversion.

## 1. Introduction

The use of solar energy to generate electricity is considered an important way to solve energy problems [1,2]. In recent years, many advanced photovoltaic energy conversion materials have been designed and developed to alleviate this energy shortage. Photovoltaic cells manufactured using photoelectric conversion materials are a popular research direction in the field of energy conversion [3]. Common photoelectric conversion materials are primarily divided into two categories: (1) inorganic materials, such as silicon [4,5], gallium arsenide [6,7], inorganic perovskite [8,9,10], and (2) organic materials, such as fullerene polymers [11,12], non-fullerene polymers [13,14], and photosensitive proteins [15,16]. Some materials exhibit great photovoltaic characteristics, for example, hybrid organohalide perovskite solar cells have achieved a power conversion efficiency of up to 23.2% [17], and non-fullerene polymers have been implemented to break the bottleneck of the fullerene-based organic solar cells with a 17.3% power conversion efficiency [18].

Photosensitive proteins, besides their high maximum theoretical potential efficiency of up to 25% [19], have many other unique advantages that give them the great potential for photovoltaic cell fabrication. For example, photosensitive proteins are abundant in various types of bacteria, and their extraction process is easy for the large-scale production [20]; as a biomaterial, their non-toxic, biodegradable, and renewable features makes them an environmentally-friendly choice, compared to other materials [21]. Photosensitive proteins have a great biocompatibility and pose no health risk to the human body [22], which gives them the great potential in medical applications, such as implantable devices.

Bacteriorhodopsin (bR) is a light-sensitive protein widely used in the field of photoelectric conversion. As the first microbial rhodopsin discovered, bR has been extensively studied for its photoelectric response and extraction method, making it an ideal choice for this study and its industry-scale application. bR is located on the cell membrane of *H. salinarum*, as a transmembrane protein with a light-driven proton pump function that transports protons from the membrane to the environment under light stimulation [23]. It is used in photoelectric conversion devices as an electrode material to construct photovoltaic cells, such as dyed sensitive solar cells (DSSCs) [24,25,26], and its photoelectric response is improved by the genetic engineering of protein and the doping of nanomaterials [27,28,29,30]. When fabricating light-sensitive energy conversion devices, the bR molecule needs to be converted into functional protein films for the bR molecules to preserve their photoelectric response functions and remain active under various environmental influences. Several techniques for the bR molecule immobilization have been implemented by researchers, including dropcasting (DC) [31], electrophoretic sedimentation (EPS) [32], Langmuir–Blodgett deposition (LBD) [33,34], affinity immobilization [35], and layer-by-layer self-assembly [36]. Each of these techniques has its advantages and disadvantages; however, the lack of relevant research and the absence of a comparison method among the different techniques, impede studies on the characteristics of bR films and delays their optimization for further industrial applications, despite the great potential of bR as a light-sensitive material.

To solve this issue, a method based on the efficiency factors for evaluating the quality of the industry-scale production of immobilized bR films, has been developed in this study. To demonstrate the feasibility of this method, three bR immobilization techniques, DC, EPS, and LBD, were implemented and the results of the immobilization were compared. The transfer of the bR molecules was verified using the absorption spectrum, with the films immobilized using the EPS technique achieving an absorption of 0.3 at 568 nm, and a topographical observation, including scanning electron microscopy (SEM) and atomic force microscopy (AFM) images, showed the successful transfer of the bR molecules. The photocurrent was measured to investigate the efficiency of the photoelectric response among the different immobilization techniques, and a novel evaluation method, the efficiency factor, was first proposed to offer a quantitative way to comprehensively evaluate different techniques. This evaluation method takes both energy conversion efficiency and immobilization time into consideration. The EPS technique has the highest efficiency factor of 13.46, which is 15.6 times that of the DC and 2.6 times that of the LBD, indicating the improvement in the energy conversion ability, due to the improved molecule orientation. These results demonstrate the excellent application of the efficiency factor in the optimization of the bR immobilization for the industry-scale production.

## 2. Result and Discussion

### 2.1. Topography and Absorption Spectrum

**Figure 1:**
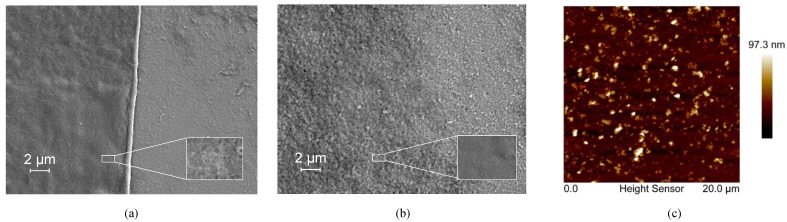
Topographical characterization of bacteriorhodopsin films immobilized with different techniques. (**a**) SEM image of the dropcasted film. A clear edge and uniformly distributed bR film indicates a good immobilization quality. (**b**) SEM image of the electrophoretic sedimented film. A clear edge and uniformly distributed bR film indicates a good immobilization quality. (**c**) AFM image of the Langmuir–Blodgett deposited film. The bR molecules are uniformly deposited on the substrate with a peak height of 97.3 nm, corresponding to three monolayers of bR.

The bR film prepared using the DC technique showed a relatively uniform morphology with no significant defects observed (Figure 1a), indicating the uniform dispersion of the bR molecules in the aqueous solution and the solid attachment of the bR molecules onto the ITO glass substrate. A clear boundary between the area covered with the bR film and the area not covered is likely the result of the “coffee ring effect” during the evaporation process of the solvent. The bR film prepared by the EPS technique showed a less clear boundary and an uneven distribution of bR fragments (Figure 1b). This is likely due to the uneven distribution of the electric field. For the bR film prepared using the LBD technique, the amount of bR molecules transferred was insufficient to be detected by SEM; therefore, the AFM imaging was used to characterize the morphology (Figure 1c). The AFM image indicates the even distribution of the bR molecules with an average surface roughness Ra = 7.58 nm, and the peak height of 97.3 nm corresponds to the three-layer bR film, indicating the successful transfer of the bR molecules.

**Figure 2:**
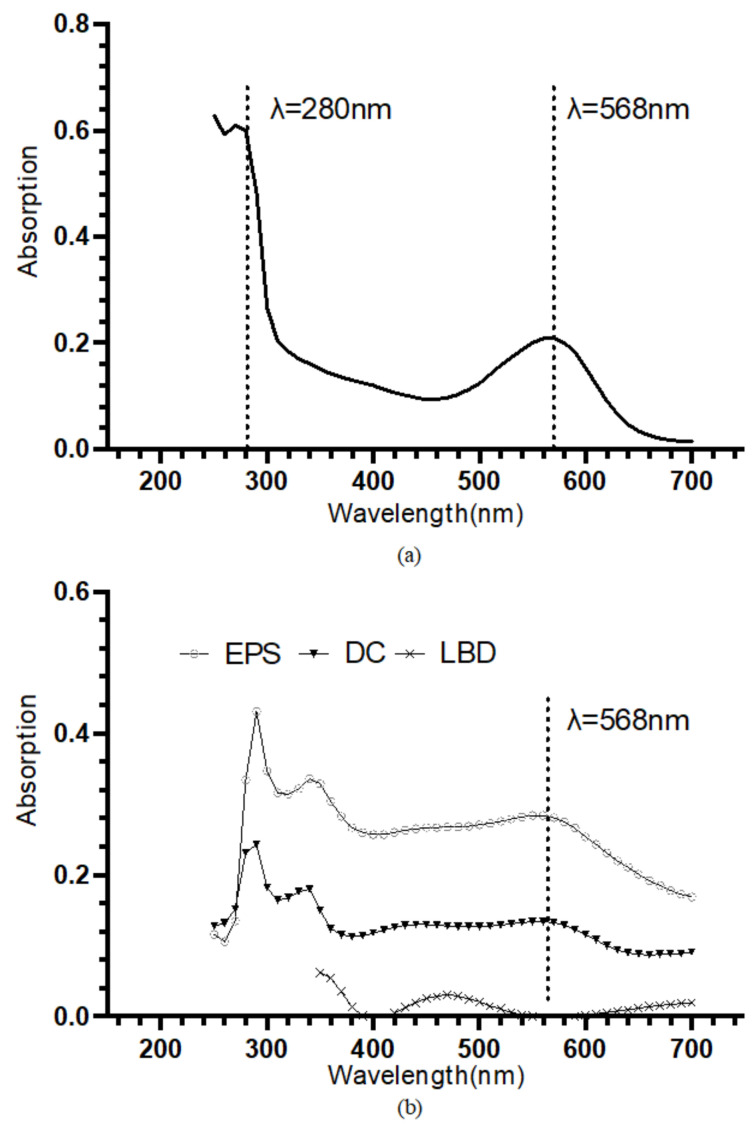
(**a**) UV-VIS absorption spectrum of the bR solution. An absorption peak at approximately 568 nm and an A_280_/A_568_ of 2.87 indicate a high purity. (**b**) UV-VIS absorption spectrum of the bR films immobilized with different immobilization techniques. Dropcasted film and electrophoretic sedimented film show the absorption peak at around 568 nm, verifying the successful transferring of bR molecule; Langmuir–Blodgett deposited film showed a considerably low absorption through the entire range, indicating a small amount of protein transferred.

The retinal contained in the bR molecules undergoes photoisomerization under illumination, which leads to the protonation and deprotonation of the Schiff base and eventually the translocation of the proton [37]. The retinal has an absorption peak at approximately 568 nm [38]. Hence the UV-VIS absorption spectroscopy is often used to specifically identify the bR molecule. The bR solution shows an absorption peak near 568 nm, and the A_280_/A_568_ of 2.87 indicates a high purity (Figure 2a). In Figure 2b, the UV-VIS absorption spectrum of the bR films immobilized using the three techniques was presented. The bR film prepared using the DC technique showed an absorption peak at 568 nm, and the bR film immobilized using the EPS technique showed an absorption peak at 562 nm, both close to that of the bR solution, indicating that the optical characteristics were well preserved after the immobilization process. The bR film immobilized using the LBD technique, however, showed a low absorption in the entire scan range, and no absorption peak was observed near 568 nm. This could be caused by the tiny amount of the bR molecule transferred to the ITO substrate: during the process of the LBD technique, most of the bR molecule was dispersed on the subphase surface instead of being transferred to the ITO substrate. This characteristic impedes the application of the LBD technique in the industrial-scale production, as the waste of raw materials is undesirable, considering the economic efficiency.

### 2.2. Photocurrent Response

The bR films generate a photocurrent under light illumination because of the light-driven proton pumping function of the bR molecule. The measurement of the photocurrents shows that the bR films prepared by the DC, EPS, and LBD techniques exhibit a photoelectric response to light stimulus. Specifically, the photocurrent was generated when the light was switched on/off, and no photocurrent was observed when the light intensity was constant, even when the bR films were continuously illuminated. The significant differential response of the bR films makes the fabrication of the biosensors for the detection of changes in the light intensity possible.

**Figure 3:**
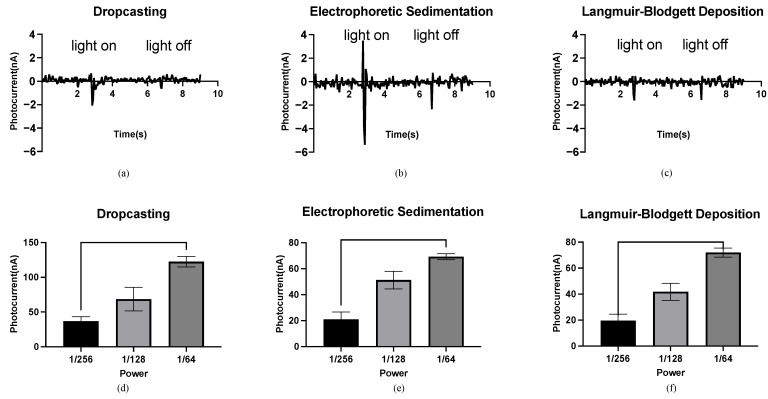
Photocurrent response of the bR films. (**a**) Photocurrent response of the bR film immobilized using the DC technique with a peak photocurrent of 2.07 nA. (**b**) Photocurrent response of the bR film immobilized using the EPS technique with a peak photocurrent of 5.38 nA. (**c**) Photocurrent response of the bR film immobilized using the LBD technique with a peak photocurrent of 1.61 nA. (**d**–**f**) Relationship between the power of the photoflash and peak photocurrent generated by the bacteriorhodopsin films immobilized with different techniques. One-way ANOVA analysis shows that the peak photocurrent increases as the power of the light source increases.

Different peak photocurrents were observed under the same light conditions for the bR films prepared using different immobilization techniques, indicating the influence of the density and orientation of the bR molecule on the photoelectric response. Density refers to the number of bR molecules per unit area of the bR film. The bR film immobilized using the DC technique shows a peak current of 2.07 nA (Figure 3a), significantly lower than 5.38 nA of the EPS technique (Figure 3b), although both methods completely transferred all of the bR molecules to the ITO glass substrate, resulting in the same density. The random orientation of the bR molecules significantly offsets the light-driven proton pumping functions under the DC technique, whereas the bR film immobilized using the EPS technique showed a higher photoelectric response, owing to the improved orientation. The negative charges of the bR molecules on the CP side are greater than those on the EC side; therefore, under the electric field, the bR molecule sediments on the cathode (in this case, the ITO glass substrate) with their CP side, are oriented toward the cathode. The pumped protons then moved from the CP to the EC side with a high efficiency, leading to a higher peak photocurrent. The bR film immobilized using the LBD technique shows the lowest peak photocurrent at 1.61 nA (Figure 3c), which is 22.2% lower than that of the bR film immobilized using the DC technique. However, the density of the bR film immobilized with the LBD technique was significantly lower than that using the DC technique (less than 10% based on estimation) because of the smaller amount of bR molecules transferred, whereas the larger surface area of the immobilized bR film indicates a higher photoelectric response efficiency for the LBD technique. These results verify the improved orientation of the bR film immobilized using the LBD technique, as the CP side of the bR molecule is more hydrophilic than the EC side. An oriented bR monolayer forms at the liquid–gas interface, and this orientation is preserved as the bR film is a typical Z-type film, with a transfer ratio close to 1 during the upward stroke and close to zero during the downward stroke.

The relationship between the light intensity and the photocurrent of the bR films is shown in Figure 3d–f. The horizontal axis represents the fraction of the maximum power (76 W) of the photoflash, and the vertical axis represents the maximum measured photocurrent. For the bR films immobilized using the DC/EPS/LBD techniques, the peak photocurrent increased with an increase in the power output, and the significant differences between the photocurrents under three levels of power output were observed. This feature of monotonicity makes the bR films an ideal material for the fabrication of light intensity detection devices, and the independence of this feature from the immobilization techniques means that the fabrication process can be implemented with more flexibility but fewer requirements.

The stability of the photovoltaic cell was demonstrated by the result of the stability test. The bR film immobilized by the EPS technique showed stability in the photoelectric response, with no significant difference in the peak photocurrent observed after three weeks (Figure S1). The proton-pumping function of the bR molecule was well-preserved after the immobilization with the solid structure. Owing to the stable photoelectric response, along with the reliable immobilization technique, the bR films have a great potential in the photosensitive device fabrication.

Moreover, the energy conversion efficiencies of the bR film and other photovoltaic materials, were compared, and the comparison result was shown in Table S1. The short circuit current *J_sc_* of the photovoltaic cells made of silicon, perovskite, and organic materials, are shown along with the peak photocurrent with the photovoltaic construct made of the bR film, immobilized using the EPS technique. Because the bR-based construct is still at its preliminary stage, many improvements can be made to improve its photoelectric response. For example, the genetic modification of the wild-type bR could alter the photocycle of the bR molecule, thus influencing the photoelectric response characteristic; the doping of nanomaterials could also improve the photosensitivity of the bR-based device by utilizing the interaction between the nanoparticle and the bR molecule, such as in the plasmonic field effect [27,29]; The implementation of the efficient electrode and the electrolyte materials will also benefit the power conversion efficiency. The bR shows a great potential as a photovoltaic material, and while a prototype has already shown the photoelectric response, an engineered device could contribute to the photoelectric performance significantly.

### 2.3. Efficiency Factor

The peak photocurrent is a crucial parameter of the immobilization techniques when the bR film is used in the fabrication of photovoltaic cells. However, other factors also should be considered when evaluating the immobilization techniques, especially when they are implemented for the industrial-scale production. Herein, we propose the following concept of the efficiency factor, which can be used as a benchmark:

(1) $$E=\frac{I_{max}\;*\;A_{total}}{T\;*\;A_{light}}$$

where E is the efficiency factor, $I_{max}$ is the peak photocurrent obtained from the photoelectric response signal recorded using the electrochemical workstation, T is the time required for the immobilization process to make a bR film, $A_{total}$ is the surface area of the immobilized film, and $A_{light}$ is the surface area under illumination. $I_{max}*\frac{A_{total}}{A_{light}}$ is the potential maximum photocurrent a bR film can generate if the entire film is illuminated. An immobilization technique with a larger efficiency factor can generate a higher potential photocurrent with a shorter immobilization time than other techniques, making it an advantageous option for the industry-scale production. When calculating the efficiency factors of different immobilization techniques, $I_{max}$ can be determined from the photoelectric response data obtained above, T varies among different immobilization techniques. The immobilization times for the DC technique and the EPS technique are 60 min and 10 min, respectively, as mentioned in the experiment section; for the LBD technique, each dipping process lasts 3 min (dipping area is 15 mm long and the dipping speed is 5 mm/min), and the total immobilization time is 15 min. In this study, $A_{total}$ for the DC and EPS techniques, is the surface area of the circle-shaped bR film with a 10 mm diameter, respectively, while for the LBD technique, $A_{total}$ is the dipping area of a 10 mm × 15 mm rectangle. $A_{light}$ is the surface area of the incident light from the spectrometer, which forms a circular shape with a 2 mm diameter. The parameters of the immobilization techniques used in this study are listed in Table 1.

According to Table 1, the ESP technique has the highest efficiency factor (=13.46) among the three immobilization techniques, which is 15.6 times that of the DC technique and 2.6 times that of the LBD technique. The high potential photocurrent originates from the improved orientation of the bR molecule, while the short immobilization time comes from the simple preparation and quick immobilization processes. The LBD technique, despite its lowest peak photocurrent, shows a higher potential photocurrent than the DC technique because of the improved bR molecule orientation owing to the different hydrophilicities of the CP and EC sides of the bR molecule.

The efficiency factor comprehensively considers the influence of the peak photocurrent, immobilization time, size of the immobilized bR film, and illumination area, making it an evaluation method to compare the immobilization techniques with a great potential. However, this evaluation method still faces many challenges under complicated industry-scale production circumstances. The equipment required and the complexity of the immobilization process vary significantly among the different immobilization techniques, which affects the cost of fabricating devices, based on bR films, the raw material cost of the unit fabrication also affects the unit cost of the bR-based devices. A photocurrent-time integral can be implemented to more accurately measure the energy conversion rate. Therefore, these parameters can be further integrated to calculate the efficiency factor when sufficient information is provided, thereby promoting the applicability and accuracy of this evaluation method.

## 3. Experimental Methods

### 3.1. Immobilization of Bacteriorhodopsin

The bR molecules (wild-type, Sigma-Aldrich, St. Louis, MI, USA, lyophilized powder) are uniformly dispersed in an aqueous solution; therefore, it can be transferred to hydrophilic substrates, such as indium tin oxide (ITO) glass. ITO glass has a high transmittance and conductivity, making it an ideal choice of substrate for the immobilization of bR. The bR molecules were dispersed in deionized (DI) water to form a 1 mg/mL solution, and then transferred to an Eppendorf tube, and sonicated for 1 min to obtain a purple transparent solution.

**Figure 4:**
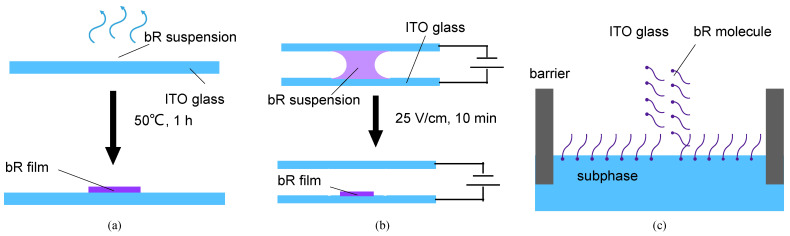
Schematics of the different immobilization techniques. (**a**) Schematics of dropcasting. Evaporation of the solvent leaves a dry bR film on the ITO glass. (**b**) Schematics of electrophoretic sedimentation. The bR molecules move toward the ITO glass under the effect of an electric field, simultaneously forming a unified orientation at the same time. (**c**) Schematics of the Langmuir–Blodgett deposition. The amphiphilicity of the bR molecules leads to an oriented monolayer on the aqueous subphase, which is then transferred onto the ITO glass substrate.

The ITO glass (transmittance = 82%, sheet resistance = 8 Ω/sq) was cleaned using ethanol and acetone for 10 min with ultrasonic vibration and then washed with DI water before use. The bR solution was dropped onto an ITO glass substrate, which was then placed in a furnace and dried at 50 °C for 80 min. A bR film was obtained after the evaporation of the water (Figure 4a).

The bR molecule is negatively charged in an aqueous solution, and its cytoplasmic (CP) side is more negative than the extracellular (EC) side at a pH > 5, and vice versa at a pH < 5 [39]. Under the influence of an electric field, the bR molecule moves towards the cathode with a consistent orientation, owing to the net electric dipole moment. The immobilization process is illustrated in Figure 4b. A 5 V DC power supply provided a consistent electric field. The ITO glass substrate and counter electrode, made of Pt foil, were connected to the cathode and anode, respectively. The bR solution, obtained using the same method mentioned above, was injected into a gap of 2 mm between the ITO glass and the Pt foil. Under an electric force, a well oriented bR film was obtained after 10 min of electrophoretic sedimentation.

The Langmuir–Blodgett deposition is a process that utilizes van der Waals forces to prepare monolayers, particularly for amphiphilic molecules. The EC side of the bR molecule is more hydrophilic on the CP side; therefore, it forms an oriented molecular layer at the liquid–gas interface with the EC side oriented toward the liquid phase [40]. Under a certain surface pressure, this monolayer is transferred to a substrate, and multiple monolayers are obtained by repeating the operation multiple times. Figure 4c shows the basic principle of this immobilization technique. The bR solution (1 mg/mL) was mixed with n-hexane and N, N-dimethylformamide (DMF) in a volume ratio of 5:5:1, and a reddish emulsion was obtained after 5 min of ultrasonication. The deposition of the bR molecule was performed using a medium-sized Langmuir–Blodgett analyzer (KSV NIMA, Biolin Scientific, Gothenburg, Sweden). The dispersed emulsion was spread on the surface of the subphase of the 0.1 M KCl solution. Following 5 min of n-hexane evaporation, the bR molecules were evenly distributed on the surface of the subphase, and the bR monolayer was squeezed at a speed of 10 mm/min. The dipping process (including upward and downward strokes) was repeated 5 times to obtain a three-layer bR film at lifting and descending speeds of 5 mm/min. The dipping was performed at a surface pressure of 25 mN/m.

### 3.2. Topographical Characterization

The absorption spectra of the bR suspension and immobilized bR films were characterized using a UV-VIS spectrophotometer (U-3900, HITACHI, Tokyo, Japan). The absorption spectra (scan range of 250–750 nm) were obtained by placing the sonicated bR suspension (concentration at 1 mg/mL) and ITO glass covered with bR films made using different immobilization techniques, into the sample chamber of the spectrophotometer.

The surface morphologies of the bR films prepared using the DC and EPS techniques were observed using SEM (Zeiss GeminiSEM 300, Oberkochen, Germany). The surface morphology was measured using an SE2 channel and a 5 kV EHT. Since the amount of bR transferred is considerably small and the SEM imaging is not sufficient to demonstrate the successful transfer of the bR molecules using the LBD technique, instead, the surface morphology was observed using AFM (Bruker Dimension icon, Billerica, MA, USA). The ITO glass substrate with the bR film was placed in the AFM sample chamber. The surface morphology was measured using a cantilever with an elasticity factor of 40 N/m in the peak-force quantitative nano-mechanics (QNM) mode.

### 3.3. Photocurrent Response Measurement

**Figure 5:**
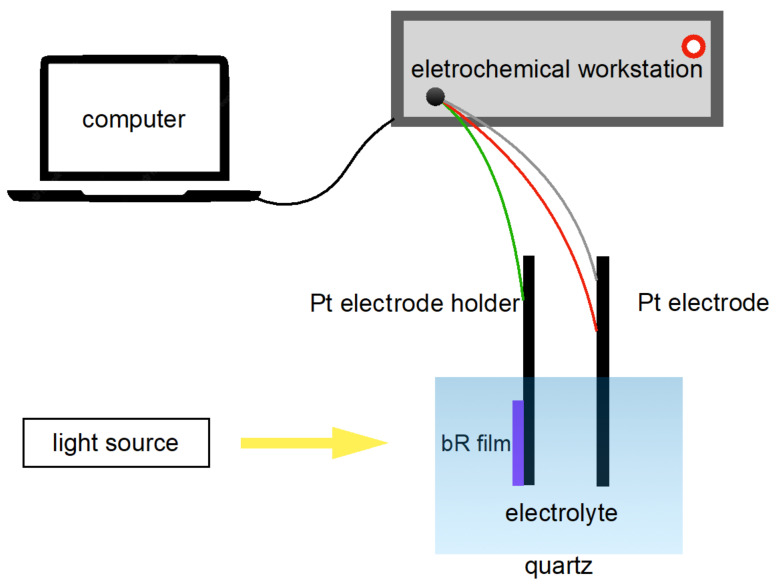
Schematics of the photocurrent measurement apparatus. The bR film initiates its proton pumping function under light stimulation, generating a photocurrent which is then detected by the electrochemical workstation and recorded by the computer.

The photocurrent response of a photocell is an important parameter for characterizing the photoelectric response of a bR film. A three-electrode photovoltaic cell was fabricated as follows: the working electrode was ITO glass covered with bR film clamped on a Pt electrode holder; the counter electrode and the reference electrode were made of Pt electrodes and the electrolyte was a mixture of 20 mM KCl and 2 mM KH_2_PO_4_ (pH = 8). Note that the photovoltaic cell was merely constructed to measure the photoelectric response of the bR films at the laboratory level and did not impose restrictions on the industry-scale production of the photovoltaic cells: other electrode materials and electrolytes can be implemented. The fabricated photovoltaic cell was connected to an electrochemical workstation (CHI660E, Shanghai Chenhua Instrument, Shanghai, China) to study its photoelectric response in an amperometric i-t mode (Figure 5).

To comprehensively understand the photoelectric response characteristics of the bR films, the influence of the immobilization techniques and incident illuminance on the photocurrents was studied. A monochromatic light source generated by a 150 W Xe lamp and a spectroscope (Omni-λ300i, Zolix instrument, Beijing, China) was implemented, and the photocurrents generated by the DC/EPS/LBD bR films under the same light conditions were compared. The relationship between the incident illuminance and photocurrent was obtained by controlling the power outputs of a photoflash (GN = 60) and measuring the peak photocurrents.

Furthermore, in order to investigate the stability of the photoelectric response of the photovoltaic cell, the peak photocurrent was measured every 48 h under the same light conditions. The peak photocurrents at day 1, day 3, and day 5 after the fabrication of the photovoltaic cell, were compared to determine whether 90% of the peak photocurrent was preserved.

## 4. Conclusions

Owing to the lack of evaluation standards for the industry-scale fabrication of bR films, we propose the concept of the efficiency factor. Three immobilization techniques were employed to demonstrate the feasibility of this evaluation method, which combines the peak photocurrent, immobilization time, and film density. Three bR immobilization techniques, DC, ESP, and LBD, were used to transfer the bR molecules to the substrates to form the functional light-sensitive bR films. The morphologies of these bR films were observed, and their photoelectric response characteristics were studied. The ESP technique showed the highest efficiency factor of 13.46, up to 16 times that of the other techniques, owing to the improved orientation of the bR molecules and the reduced immobilization time. The bR film immobilized using the EPS technique also exhibits a high stability and sensitivity, with no significant decline in the peak photocurrents three weeks after the photovoltaic cell fabrication, and a photocurrent density of 1.71 µA/cm^2^, detected even under the incident light of power lower than 0.01 mW/cm^2^. These characteristics make the EPS technique an advantageous solution for the industry-scale fabrication of the bR-based solar cells. The efficiency factor, proposed as a novel evaluation method, showed a great feasibility in this research for comparison and optimization of the bR immobilization techniques. While the industry-scale application of the bR film is still at its preliminary stage, this research contributes to providing immobilization techniques with a high energy conversion efficiency and a low cost.

The bR-based photovoltaic construct in this study has many advantages. The EPS fabrication process is simple, fast, and easy to implement, as it requires no specialized equipment, the bR as a type of biomaterial provides a pollution-free, non-toxic, environmentally-friendly alternative to other photosensitive materials, and the high biocompatibility of bR films offers a great potential in medical applications. While some limitations, due to the lack of relevant research, still exist, many improvements can be made with support from further studies. The implementation of novel electrode/electrolyte materials can benefit the power conversion efficiency, and the photocycle of bR can be shortened by the genetic engineering or the doping of nanomaterials, to increase the photoelectric response. The bR film has already shown a great potential in the solar energy conversion field, and further engineering of bR-based devices could contribute to its performance enhancement and industry-scale application.

## Figures and Tables

**Table 1:** Efficiency factors of the different immobilization techniques.

Immobilization Technique	Dropcasting	Electrophoretic Sedimentation	Langmuir–Blodgett Deposition
*I_max_*/nA	2.07	5.38	1.61
*T*/min	60	10	15
*A_total_*/mm^2^	78.54	78.54	150
*A_light_*/mm^2^	3.14	3.14	3.14
*E*	0.86	13.46	5.13

## Data Availability

Data is contained within the article or Supplementary Materials.

## Supplementary Materials

The following supporting information can be downloaded at: https://www.mdpi.com/article/10.3390/ijms232416079/s1. References [5,17,18] are cited in the Supplementary Materials.

## Abbreviations

AFM: atomic force microscopy; ITO, indium tin oxide; SEM, scanning electron microscopy.
