# Supplementary material for: Engineered Bacteriorhodopsin Film with Oriented Patterns for the Improvement of the Photoelectric Response
Source: Int J Mol Sci. 2022 Dec 16;23(24):16079. doi: 10.3390/ijms232416079 (PMC9785767; doi:10.3390/ijms232416079)
Supplement: Supplementary file 1 [file ijms-23-16079-s001.zip › ijms-2026674-supplementary.pdf]

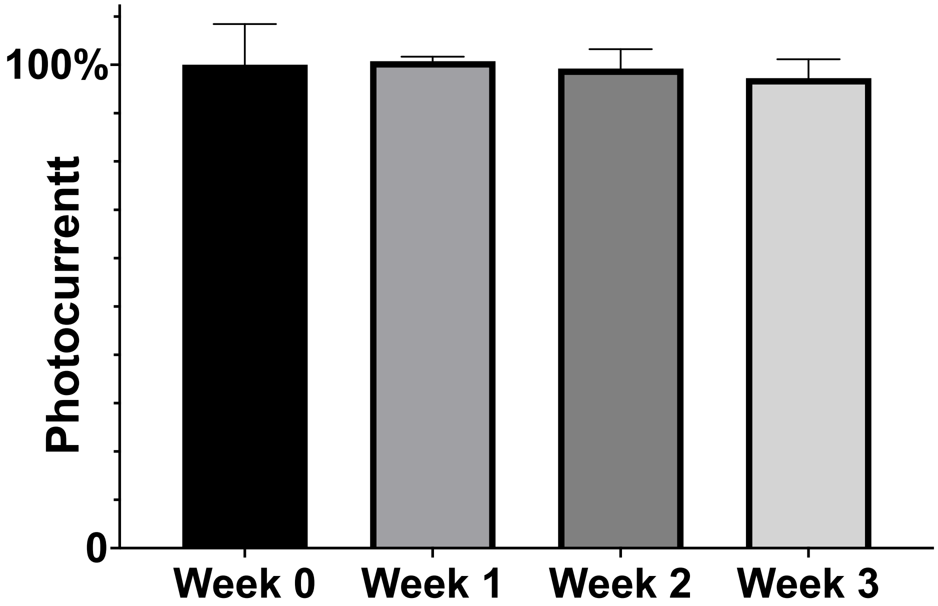

Figure. S1. Stability of the photoelectric response of the bR-based photovoltaic cell. The peak photocurrents were measured on weeks 0, 1, 2, and 3, and are shown in the figure as the percentage of the mean photocurrent from week 0. ANOVA analysis showed that $p=0.8499>0.5$, indicating no significant difference among peak photocurrents measured at different time, demonstrating the stability of the bR-based photovoltaic cell.

Table S1. Photoelectric response of photovoltaic cells made of different materials

Material	Light intensity	J_{sc}/peak photocurrent	Reference
c-Si with TPC	One-sun (100 mW/cm^2)	40.87 mA/cm^2	[5]
Perovskite with PEAI treatment	One-sun (100mW/cm^2)	24.9 mA/cm^2	[17]
Organic tandem cell	One-sun (100mW/cm^2)	14.35 mA/cm^2	[18]
EPS wild-type bR film	< 0.01mW/cm^2	1.71 μA/cm^2	this work

Note: the light intensity on the EPS wild-type bR film was calculated by measuring the power of light after going through the ITO glass substrate without the bR film and subtracting the power of light after going through the ITO glass substrate with the bR film. The exact value cannot be determined owing to the limit of the measuring device.
